# Supplementary material for: 3D oxygen vacancy distribution and defect-property relations in an oxide heterostructure
Source: Nat Commun. 2024 Jun 26;15:5400. doi: 10.1038/s41467-024-49437-0 (PMC11208508; doi:10.1038/s41467-024-49437-0)
Supplement: Supplementary file 1 — Supplementary Information [file 41467_2024_49437_MOESM1_ESM.pdf]

Supplementary Information

3D oxygen vacancy distribution and defect-property relations in an oxide heterostructure

Kasper A. Hunnestad[1,9,10], Hena Das[2], Constantinos Hatzoglou[1], Megan Holtz[3,4], Charles M. Brooks[4], Antonius T. J. van Helvoort[5], David A. Muller[3,6], Darrell G. Schlom[4,6,7], Julia A. Mundy[8], Dennis Meier[1]*

[1]Department of Materials Science and Engineering, NTNU Norwegian University of Science and Technology, 7491 Trondheim, Norway

[2]Institute of Innovative Research, WRHI, Tokyo Institute of Technology, 4259 Nagatsuta, Midori-ku Yokohama 226-8503, Japan

[3]School of Applied and Engineering Physics, Cornell University, Ithaca, New York 14853, USA

[4]Department of Materials Science and Engineering, Cornell University, Ithaca, New York 14853, USA

[5]Department of Physics, NTNU Norwegian University of Science and Technology, 7491 Trondheim Norway

[6]Kavli Institute at Cornell for Nanoscience, Ithaca, New York 14853, USA

[7]Leibniz-Institut für Kristallzüchtung, Max-Born-Str. 2, 12489 Berlin, Germany

[8]Department of Physics, Harvard University, Cambridge, Massachusetts 02138, USA

[9]Current address: Acoustics Group, Department of Electronic Systems, NTNU Norwegian University of Science and Technology, 7491 Trondheim, Norway;

[10]Current address Centre for Geophysical Forecasting, NTNU Norwegian University of Science and Technology, 7491 Trondheim, Norway.

*Corresponding author: dennis.meier@ntnu.no

Supplementary Figures

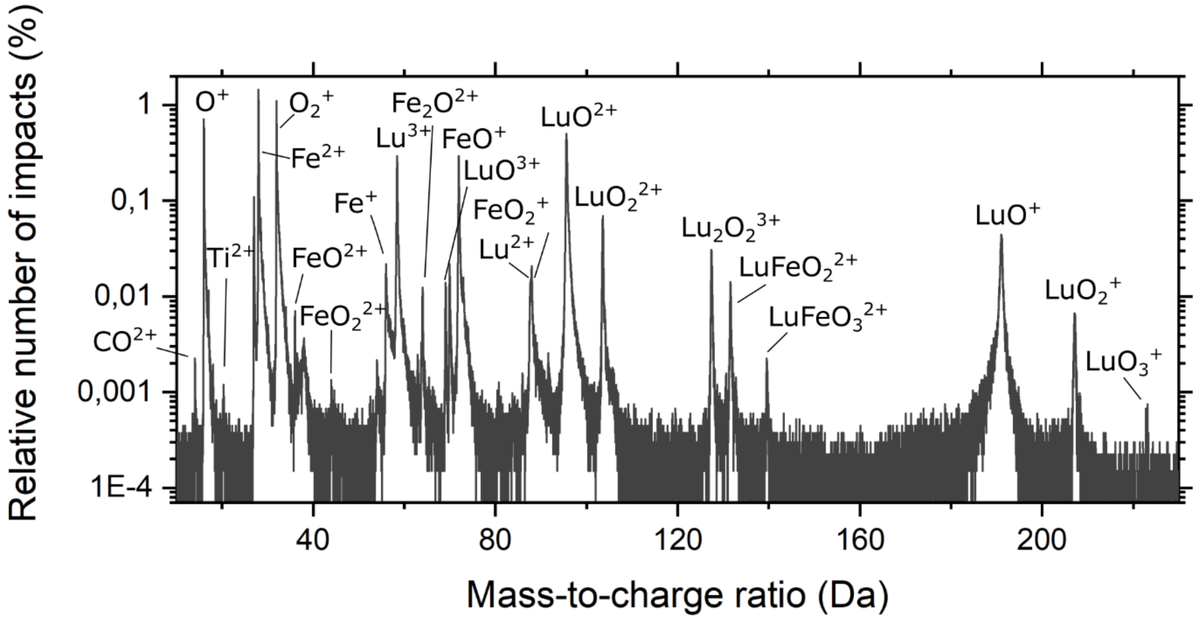

Figure S1: Mass spectrum of the $(LuFeO_3)_9/(LuFe_2O_4)_1$ superlattice. Figure shows the mass spectrum of the superlattice shown in Figure 1 in the main text. The spectrum is mainly coming from the thin film, but minor peaks from the substrate and capping layer are present, such as Ti^{2+}, which originates from the Ti protection layer. Species coming from the substrate are not labelled. In cases where peaks from the substrate and thin film overlap, the peak is assigned to the species belonging to the thin film. By integrating over the peaks, the average chemical composition from the thin film is found to be 53.74 ± 0.04 at.% O, 23.78 ± 0.05 at.% Lu and 22.56 ± 0.05 at.% Fe.

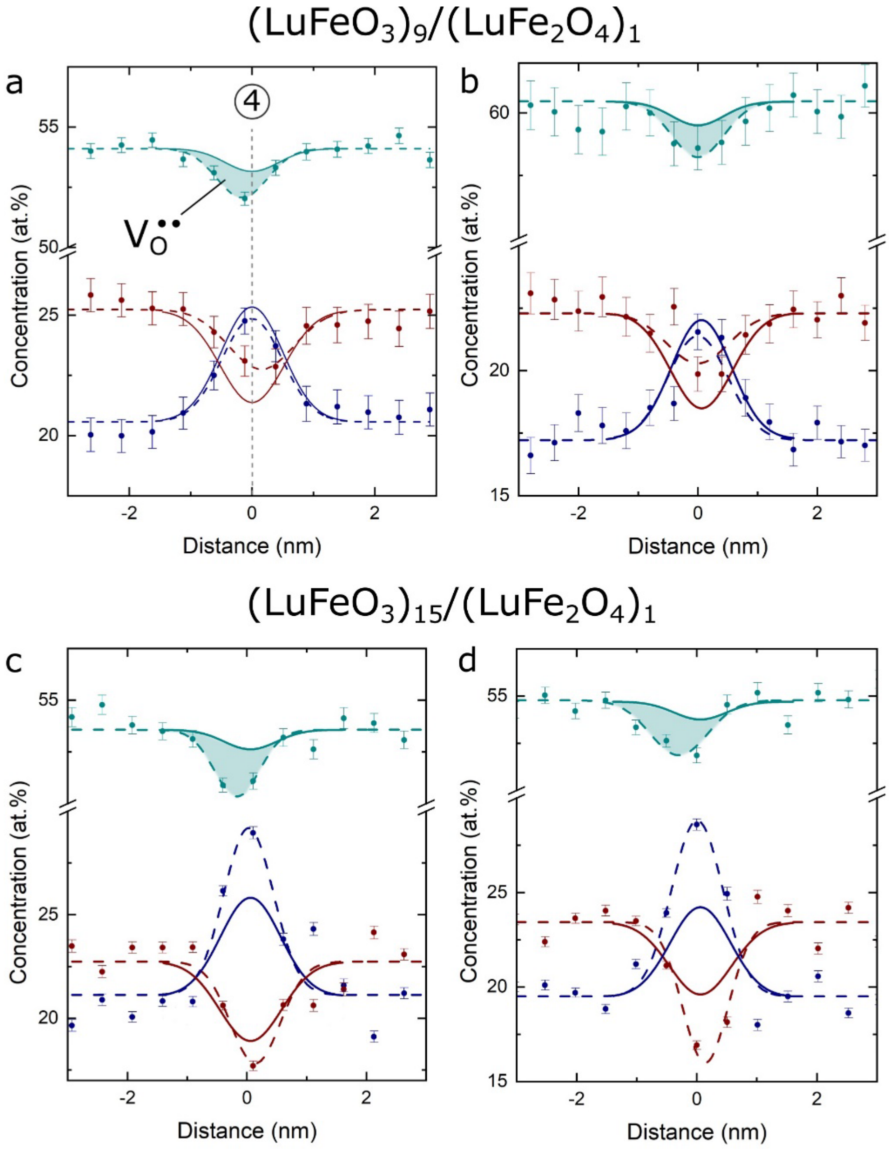

Figure S2: APT analysis of additional $(LuFeO_3)_m/(LuFe_2O_4)_1$ superlattices. a and **b,** APT analysis of additional double-Fe layers in the $(LuFeO_3)_9/(LuFe_2O_4)_1$ superlattice. Peak ④ from Figure 2a is shown (dashed line is a gaussian fit) and compared to a simulation (solid line) of the expected concentration profile from the superlattice. The deviation in O concentration (highlighted by the shaded area) indicates an oxygen vacancy density (v_O) of about (5.6 ± 1.7) x 10^{13}/cm^2. **b**, Shows the compositional profile from an additional sample, whereas **c** and **d** shows the compositional profiles from a $(LuFeO_3)_{15}/(LuFe_2O_4)_1$ superlattice sample instead, grown under similar conditions. All data sets consistently show oxygen depletion compared to the DFT-based models, corresponding to an accumulation of oxygen vacancies, accompanied by fluctuations in the Fe/Lu ratio.

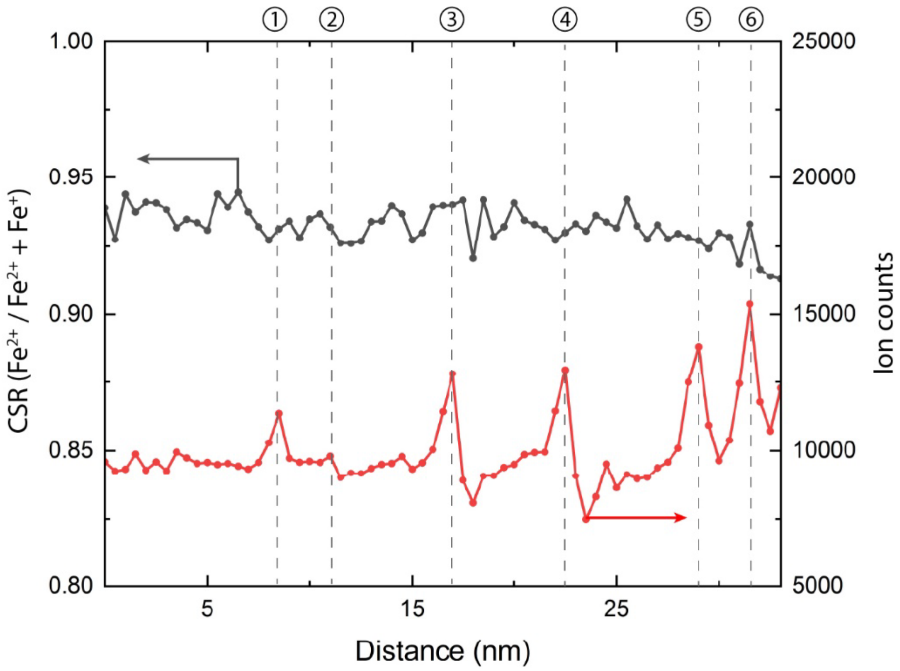

Figure S3: **Charge-state ratio (CSR) analysis.** CSR analysis of the superlattice system, using the Fe charge state (black line). The dashed lines indicate the position of the $LuFe_2O_4$ layers, obtained by considering the ionic density profile (red line), which can detect very subtle changes to the electric field evaporation criteria. Importantly, the CSR analysis does not identify any substantial changes to the electric field strength, which rules out any major artefacts stemming from changes to the field evaporation criteria.
